# Recovering Hidden Responder Groups in Individuals Receiving Neurofeedback for Tinnitus

**DOI:** 10.3389/fnins.2022.867704

**Published:** 2022-06-23

**Authors:** Constanze Riha, Dominik Güntensperger, Tobias Kleinjung, Martin Meyer

**Affiliations:** ^1^Department of Psychology, University of Zurich, Zurich, Switzerland; ^2^Research Priority Program “ESIT—European School of Interdisciplinary Tinnitus Research,” Zurich, Switzerland; ^3^Department of Otorhinolaryngology, University Hospital Zurich, Zurich, Switzerland; ^4^Neuroscience Center Zurich (ZNZ), ETH Zürich, Zurich, Switzerland; ^5^University Research Priority Program “Dynamics of Healthy Aging,” University of Zurich, Zurich, Switzerland

**Keywords:** tinnitus, neurofeedback (NFB), inefficacy problem, EEG, brain computer interface, growth mixture model, responder, heterogeneity

## Abstract

The widespread understanding that chronic tinnitus is a heterogeneous phenomenon with various neural oscillatory profiles has spurred investigations into individualized approaches in its treatment. Neurofeedback, as a non-invasive tool for altering neural activity, has become increasingly popular in the personalized treatment of a wide range of neuropsychological disorders. Despite the success of neurofeedback on the group level, the variability in the treatment efficacy on the individual level is high, and evidence from recent studies shows that only a small number of people can effectively modulate the desired aspects of neural activity. To reveal who may be more suitable, and hence benefit most from neurofeedback treatment, we classified individuals into unobserved subgroups with similar oscillatory trajectories during the treatment and investigated how subgroup membership was predicted by a series of characteristics. Growth mixture modeling was used to identify distinct latent subgroups with similar oscillatory trajectories among 50 individuals suffering from chronic subjective tinnitus (38 male, 12 female, mean age = 47.1 ± 12.84) across 15 neurofeedback training sessions. Further, the impact of characteristics and how they predicted the affiliation in the identified subgroups was evaluated by including measures of demographics, tinnitus-specific (Tinnitus Handicap Inventory) and depression variables, as well as subjective quality of life subscales (World Health Organization—Quality of Life Questionnaire), and health-related quality of life subscales (Short Form-36) in a logistic regression analysis. A latent class model could be fitted to the longitudinal data with a high probability of correctly classifying distinct oscillatory patterns into 3 different groups: non-responder (80%), responder (16%), and decliner (4%). Further, our results show that the health-related wellbeing subscale of the Short Form-36 questionnaire was differentially associated with the groups. However, due to the small sample size in the Responder group, we are not able to provide sufficient evidence for a distinct responder profile. Nevertheless, the identification of oscillatory change-rate differences across distinct groups of individuals provides the groundwork from which to tease apart the complex and heterogeneous oscillatory processes underlying tinnitus and the attempts to modify these through neurofeedback. While more research is needed, our results and the analytical approach presented may bring clarity to contradictory past findings in the field of tinnitus research, and eventually influence clinical practice.

## Introduction

Chronic tinnitus is a variable phenomenon characterized by a heterogeneous appearance (Cederroth et al., 2019). Existing data suggest an extensive degree of individual differences and fluctuations in tinnitus, which have hampered both basic and clinical research (Hall et al., 2018). Previous research has aimed to disentangle the complex heterogeneity of the audiological phantom percept into causal risk factors, such as gender, age, ototoxic medication, and related hearing loss (Davis and El Refaie, 2000; Martines et al., 2010) or tinnitus characteristics (e.g., loudness, pitch, side of perception, and duration). Further presumed causes of the heterogeneity are comorbidities, which may start with anxiety (McCormack et al., 2015) or insomnia (Lasisi and Gureje, 2011; Wallhäusser-Franke et al., 2013), continue to hyperacusis (Goebel and Floezinger, 2008), and escalate to depression (McKenna et al., 1991; Zöger et al., 2006; Zirke et al., 2013; Trevis et al., 2018). Other epiphenomena, including tinnitus-related distress (Hesser and Andersson, 2014; Brüggemann et al., 2016), personality traits (Konareva, 2006; Simões et al., 2019), and tinnitus-specific brain oscillation accompanied with structural and functional alterations in auditory and non-auditory brain areas (Schlee et al., 2009; Adjamian et al., 2014) have been considered to contribute to or moderate the various manifestations of the phantom percept. Finally, all possible combinations of the mentioned phenomena complement the heterogeneous appearance (Henry et al., 2005; Vanneste et al., 2010; Joos et al., 2012; Vanneste and De Ridder, 2012; Meyer et al., 2014). As efforts to disentangle the heterogeneity have increased, so has the recognition of the complexity of an effective treatment approach (Scott and Lindberg, 2000; Hoare et al., 2011; Pryce et al., 2019).

A treatment approach for such a heterogeneous phenomenon that is appropriate for all those suffering from tinnitus—a “one size fits all” solution, so to speak—has not yet been identified (Landgrebe et al., 2012; Baguley et al., 2013; Hall et al., 2019). The difficulty arises as the heterogeneous appearance of tinnitus persists in its response to treatment with complex and variable trajectories (Tyler et al., 2008; Hall et al., 2016; Riha et al., 2021). However, a group of treatment modalities has recently inspired extensive research; namely, neurofeedback (NFB) (Guerra et al., 2019). In most cases, this technique offers a non-invasive window on the brain and provides a tool to pinpoint and alter subject-specific brain function and dysfunction, thus offering potential for improvement of a number of (clinical) conditions, such as ADHD, depression, epilepsy, and anxiety, among others (for an overview, see Hampson et al., 2019). In the treatment of tinnitus, NFB training has been associated with reductions in self-reported tinnitus-related distress and loudness (Dohrmann et al., 2007a,b; Crocetti et al., 2011; Güntensperger et al., 2019; Jensen et al., 2020). The underlying mechanism of NFB is based on the reinforcement of individual brain activity patterns that are recorded *via* electroencephalography (EEG) or functional magnetic resonance imaging (fMRI), analyzed and fed-back to the participant in real time. The feedback modality can be either visual, acoustic, or tactile and is based on the principles of operant conditioning (Skinner, 1938). The participant is rewarded when the brain signal reaches a predefined value. Despite the great potential of NFB as an option in the treatment for several conditions, the practical application still encounters considerable drawbacks.

One potential source of drawback is the general ability of an individual to modify their cortical activity, which is referred to as the *inefficacy problem* (The inefficacy problem is apparent in both EEG- and fMRI-based NFB; yet rooted in diverse technical approaches and difficulties. Gevensleben et al., 2009; Weber et al., 2011; Huster et al., 2014; Rogala et al., 2016; Alkoby et al., 2018). This failure to control has been described in numerous NFB trials and other brain-computer interface (BCI) applications (for a review, see Alkoby et al., 2018). Alkoby et al. (2018) note that in most NFB studies approximately 16–57% of the participants are successful in self-regulating their EEG activity. A further consideration is that there is no consensus yet about how to quantify effectiveness, thus the definition of a responder is not consistent within the NFB and BCI literature and still lacks a general standard across studies and research fields (Gruzelier, 2014a,b). In addition, the question has been raised whether the ability to deliberately modify the oscillatory activity is necessarily linked to the NFB training outcome, or vice versa; for example, the reduction of symptoms such as tinnitus distress and loudness (Rogala et al., 2016). Existing evidence indicates that generally the outcome of NFB treatment is related to combined effects of pre-treatment, neuroanatomical or oscillatory, and treatment-specific factors. Among the pre-treatment factors are age and sex (Riha et al., 2021), personality traits (Simões et al., 2019), and psychological factors such as motivation (Diaz Hernandez et al., 2018), mood, attention, and anxiety (Koush et al., 2017), which further influence different (oscillatory) baseline conditions for NFB training. For a systematic review of how psychological factors contribute to NFB outcome, we refer the reader to Kadosh and Staunton (2019). Baseline neuroanatomical or oscillatory determinants have included gray and white matter volumes (Enriquez-Geppert et al., 2013; Ninaus et al., 2015), as well as the means of eyes-open resting-state EEG power before the training (Wan et al., 2014; Nan et al., 2015; Reichert et al., 2015). Further factors in the design of the training protocol (e.g., duration of each training and training schedule), and the NFB learning strategy (Kober et al., 2013; Witte et al., 2013) may contribute to overall NFB success. Indeed, the evaluation of early training sessions can be used to predict future training progress (Weber et al., 2011; Enriquez-Geppert et al., 2013). In addition to those already mentioned, Weber et al. (2020) have provided an extensive summary of predictors of NFB training outcome in their systematic review. However, even this is not an exhaustive list, and the conflicting results provided only underscore the need for further research in the field of NFB training.

For the present research and in the light of the *inefficacy problem*, the first question was whether the tinnitus individuals studied were able to alter their brain activity in the predefined direction. If the desired change in neural activity was apparent across the NFB training, the individual is considered a *Responder* in this report, independent of tinnitus-related changes. Due to the pronounced variation of the oscillatory fingerprint in tinnitus and the variation in the response to NFB, the main purpose of this study was to identify unobserved subgroups of individuals that had similar EEG training trajectories across all sessions. By disentangling the heterogeneity of training trajectories into subgroups, we further investigated which potentially modifiable clinical factors predicted group affiliation *prior* to the NFB training. Thus, we aimed at identifying the underlying characteristics that were associated with successful oscillatory modification, and thereby recognizing the possible Responders to NFB. This research thus constitutes the conceptual groundwork for identifying subgroups of individuals that are more or less responsive to the given intervention, in the sense of being able to alter one’s brain activity. Further, it contributes to the understanding of inter-individual differences in NFB progress, knowledge which may then be applied in the development of individually tailored NFB protocols with the aim of increasing the therapy’s effectiveness.

## Materials and Methods

### Study Sample

The study sample in this analysis was derived from the clinical trial by Güntensperger et al. (2017, 2020, 2019), the largest NFB study in tinnitus research to date. The authors’ main goal was to examine the efficacy and possible distinctions of two different NFB approaches in the treatment of tinnitus; namely, traditional surface-based NFB vs. tomographic NFB (Güntensperger et al., 2020). The protocol complied with the Declaration of Helsinki, was approved by the relevant Ethics Committee (Kantonale Ethikkommission Project KEK-ZH-Nr. 2014-0594), and further registered online at ClinicalTrials.gov (NCT02383147) and kofam.ch (SNCTP000001313). The trial took place in 2017 and 2018, and comprised two baseline visits, 15 weekly NFB sessions of 15 min duration each, a post-treatment visit, as well as two follow-up appointments 3 and 6 months after completion of training. Fifty individuals with chronic tinnitus were able to complete the NFB study, including 38 males and 12 females aged 47.1 ± 12.84 (*M* ± *SD*) years (Güntensperger et al., 2019, 2020). Each participant gave their written informed consent prior to partaking in the experimental trials. We refer to the original referenced publication for an in-depth description of the study protocol, and the ancillary publication by Riha et al. (2021) for properties of applied measures and their predictive value regarding the progression of NFB training. Table 1 provides an overview of characteristics, health, and tinnitus characteristics of the study sample.

**TABLE 1:** Demographic, health, and tinnitus characteristics of study sample.

		Mean	*SD*	Median	Min	Max
	Age	47.10	12.84	46.00	24.00	75.00
	Mean hearing loss (dB)	7.32	8.80	4.15	0.00	34.40
	Tinnitus duration (months)	110.12	126.43	49.00	8.00	720.00
Tinnitus &	THI	33.64	17.72	30.00	4.00	84.00
Depression	BDI	6.40	5.02	5.50	0.00	22.00
	SCL	0.67	0.57	0.50	0.00	2.67
SF-36	Physical functioning index	5.70	9.79	0.00	0.00	50.00
	Role-physical index	17.00	26.46	0.00	0.00	100.00
	Bodily pain index	16.16	21.58	0.00	0.00	79.00
	General health perceptions index	29.10	17.15	28.00	0.00	65.00
	Vitality index	46.80	15.28	45.00	20.00	80.00
	Social functioning index	15.00	18.39	12.50	0.00	62.50
	Role-emotional index	18.00	31.01	0.00	0.00	100.00
	Mental Health index	32.24	15.51	30.00	0.00	68.00
WHO-QoL	Physical	77.71	13.47	78.57	42.86	100.00
	Psychological	71.42	14.94	75.00	33.33	95.83
	Social	68.33	18.75	70.83	25.00	100.00
	Environmental	82.94	12.57	85.94	46.88	100.00

*THI, Tinnitus Handicap Inventory; BDI, Beck Depression Inventory; SCL, Symptom Checklist; WHO-Qol, World Health Organization Quality of Life Questionnaire; SF-36, Short Form Health Questionnaire; SD, standard deviation.*

### Brain Oscillation and Tinnitus

A common finding in brain imaging resting-state EEG studies of patients suffering from chronic tinnitus is an increased delta (3–4 Hz) wave activity and a reduction in alpha (8.5–12 Hz) oscillation in the auditory cortex region compared to healthy subjects (Weisz et al., 2007a,b, 2011; De Ridder et al., 2015). In chronic tinnitus, the cause of these established, spontaneous oscillatory alterations has been linked to sensory deprivation; namely, deafferentation due to hearing loss (Llinás et al., 1999; Eggermont and Roberts, 2004; Møller, 2007; Weisz et al., 2007b; Eggermont, 2012). The consequence of these bottom-up and top-down abnormalities is an imbalance in excitatory-inhibitory neuronal activity along the tonotopic axis in the affected regions (Møller, 2007; Hong et al., 2016). Among others, such adaptions are described in the theoretical frameworks of the thalamocortical dysrhythmia model (TCM; Llinás et al., 1999; Mahmoudian et al., 2013) and the synchronization by loss of inhibition model (SLIM; Weisz et al., 2007b). Thus, a frequently used NFB training protocol for tinnitus aims to reduce delta and increase the individual alpha activity to attenuate tinnitus and tinnitus-related symptoms (Dohrmann et al., 2007b; Crocetti et al., 2011; Güntensperger et al., 2019, 2020; Jensen et al., 2020). Using a rewarding alpha and inhibiting delta protocol resulted in encouraging training outcomes in previous NFB trials. For an overview of this and other NFB protocols in the treatment of tinnitus (see Güntensperger et al., 2017).

**FIGURE 1:**
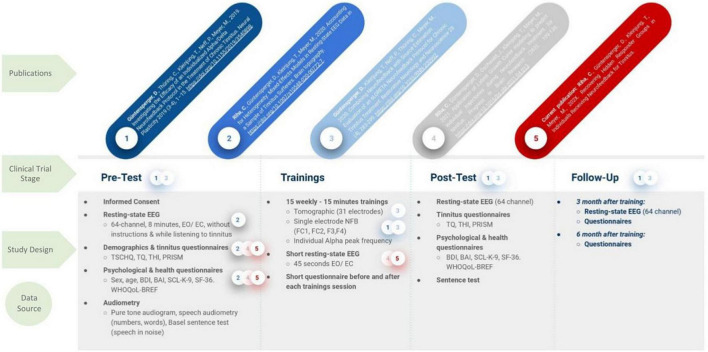
Study Design of the clinical NFB trial by Güntensperger et al. (2019, 2020). Based on the original data a number of publications evolved, however, focusing on different aspects of the data set, according to the hypotheses. The used data sets are highlighted by a color coded circle with a number, which correspond to the publications in the first row. TSCHQ, Tinnitus sample case history questionnaire; THI, Tinnitus Handicap Inventory; TQ, Tinnitus Questionnaire; PRISM, Pictorial Representation of Illness and Self Measure; BDI, Beck Depression Inventory; SCL K 9, Symptom Checklist; WHO, World Health Organization Quality of Life; SF 36, Short Form Health Questionnaire.

Güntensperger’s NFB trial from 2017 to 2018 applied the previously described protocol who additionally acquired the neuro-dynamic data for this analysis as mentioned before (Güntensperger et al., 2019, 2020). The measures used in this report were the EEG power values from alpha and delta, recorded with fronto-central electrode positions (FC1, FC2, F3, F4) *prior* to each of the 15 NFB training sessions (thus unrelated to the training itself). The resting-state activity was recorded by splitting it in eyes-closed and -open segments, whereby we focused on the latter according to the recommendations of the European tinnitus research network, TINNET (Working Group 3).^1^ The EEG data derived from each of the 15 recordings was pre-processed and the EEG power averaged for each participant across the four electrodes, according to the main interest in this analysis, the individual trajectories. In order to examine the individual training trajectories, the ratio between the desired increase of alpha and decrease of delta power (alpha/delta ratio; ADR) was calculated and compared across time points. The interested reader is referred to publications by Güntensperger et al. (2019, 2020) and Riha et al. (2020, 2021) for in-depth descriptions of the EEG recording procedure and pre-processing pipelines. To resolve the possible confusion around the different publications evolving from this data set we further refer to Figure 1 for a comprehensive overview.

### Analytical Procedure

#### Oscillatory Training Trajectories

This analysis follows on from a previous analysis, in which we investigated the oscillatory trajectories of delta and alpha and their relation to influential factors across the NFB training (Riha et al., 2021). The applied latent growth curve (LGC) analysis revealed a linear pattern of change and a significant individual variability in the two frequency bands over time: The desired enhancement of alpha was found, while slow wave delta was stable in most individuals throughout the NFB training. These results raised questions that inspired this explorative follow-on analysis with the aim of identifying unobserved subgroups (latent classes) with similar ADR patterns in the variability of longitudinal linear trajectories.

**FIGURE 2:**
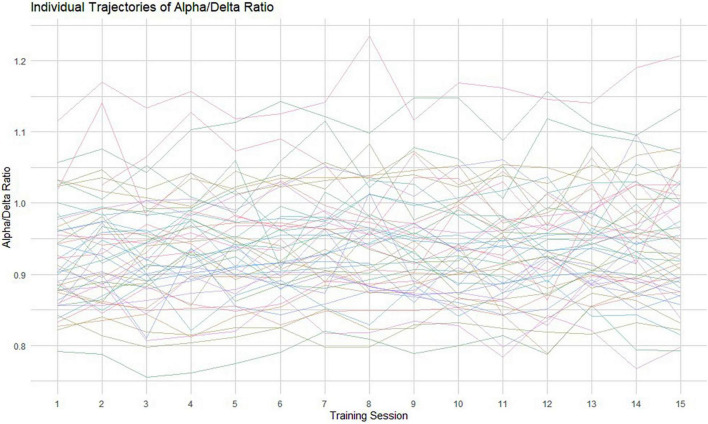
Individual raw data trajectories of the ADR across the 15 measurement occasions. Each thin line represents an individual oscillatory ADR trajectory.

Here, we used a growth mixture modeling (GMM) approach (Muthen and Muthen, 2000; Kaplan, 2004; Muthén, 2004; Jung and Wickrama, 2008; Ram and Grimm, 2009; Berlin et al., 2014; Geifman et al., 2018) to statistically differentiate meaningful or naturally occurring subgroups according to the trends in repeated measures of the ADR (see Figure 2 for the individual ADR trajectories). In simple terms, by including the categorical variable of “class,” the GMM approach is able to determine the optimal number of classes, the number of people in each class, as well as the growth factors (intercept and slope) of each different trajectory. We employed an exploratory approach and fitted models with an increasing number of classes to ascertain the optimum latent class model. To estimate the number of latent classes, we followed recommended approaches including the comparison of various model fit statistics, substantive meaning and interpretability of each class (Wickrama et al., 2016). We inspected the Akaike (1974) and the Bayesian information criterions (BIC; Schwarz, 1978), the sample-size adjusted BIC (SSABIC; Sclove, 1987), entropy values and the Lo-Mendel-Rubin likelihood ratio test value (LMR-LRT; Jung and Wickrama, 2008). For interpretation, lower AIC, BIC, and SSABIC values indicate a more parsimonious and better fitting model, whereas higher entropy values signal better class separation (Nylund et al., 2007). Models were estimated by full maximum likelihood (FML) and robust standard errors (MLR) to non-normality and non-independence of observations.

#### Class Membership

In a second step and since latent classes (i.e., the identified subgroups) are categorical, we applied Firth’s logistic regression (Firth, 1993) with the penalization of log-likelihood (Heinze, 2006) to estimate the association with a list of characteristics in a small sample (Heinze and Puhr, 2010). This kind of logistic regression is designed to handle datasets that are small, imbalanced or separated. The estimates represent the logarithm of the odds of being in a latent class vs. being in the reference class, while assessing the overall model fit and predictive accuracy. Moreover, we reported the Nagelkerke *R*^2^ and Hosmer and Lemeshow test value as quality markers for this analysis. For the model’s diagnostic properties of sensitivity and specificity, we used the receiver operating characteristic (ROC) curve and the area under the curve (AUC) as a measure of predictive ability. The list of characteristics that we considered to possibly mark class membership were acquired face-to-face during the two baseline visits, and include age, sex, tinnitus duration in months, as well as scores from a tinnitus-related symptom scale (THI: Tinnitus Handicap Inventory—German version; Kleinjung et al., 2007) and depression scales (BDI: Beck Depression Inventory; Hautzinger et al., 1994); SCL-K-9: short form of the Symptom Checklist—(Klaghofer and Brähler, 2001). Additionally, the subscales of the Quality of Life questionnaire from the World Health Organization (WHOQOL: World Health Organization Quality of Life-BREF—German version; Angermeyer et al., 2002) and the health-related questions from the Short Form-36 (SF-36—German version; Bullinger et al., 1995) were possible indicators. For a more detailed description of this list of characteristics, we refer to our preceding analysis (Riha et al., 2021). Further, the complete test battery used in the clinical study by Güntensperger and colleagues followed the guidelines of the Tinnitus Research Initiative (TRI; Landgrebe et al., 2012).

For reasons of completeness, we included the categorical feature of sex, and encoded it as dichotomous (0 = female; 1 = male). Questionnaire items in the logistic regression were treated as continuous measures and were mean-centered prior to the analysis (Hox, 2002). *P*-values below 0.05 were considered to indicate statistical significance. All analysis was performed using R statistical software, version 4.0.0 (R Core Team, 2019). The following packages were used: “lcmm” for the growth mixture model analysis (Proust-Lima et al., 2017), the “logistf” package for Firth’s logistic regression (Heinze and Ploner, 2004), and plots were created using “ggplot2” (Wickham, 2016).

## Results

**FIGURE 3:**
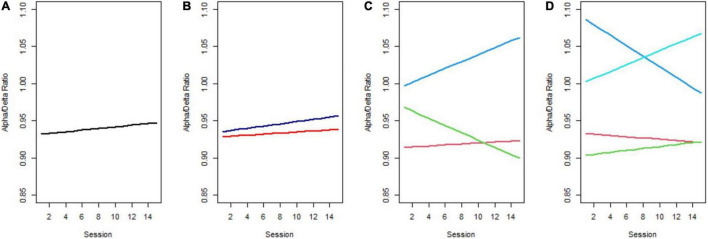
Predicted trajectories across 15 NFB training sessions. **(A)** 1-Class model, **(B)** 2-class model, **(C)** the favored 3-class model, with Class 1 corresponding to the red, Class 2 to green, and Class 3 to the blue line, **(D)** 4-class model.

The considerable individual variability in the ADR trend among all tinnitus sufferers across the NFB training is shown in Figure 2 (raw data). To ensure that we identified the model of change that best represented the 15 training sessions, we conducted a GMM analysis. This approach was chosen to extract unobserved subgroups of tinnitus sufferers with homogenous change trajectories. In Figure 3 the predicted means of the 1-, 2-, 3-, and 4-class model can be compared. Additionally, Table 2 provides the AIC, BIC, SSBIC values and the entropy results for the estimated class models. The explorative model fitting procedure resulted in a 3-class model, and the decision was facilitated by the recommendation for fit indices. The 3-class model is favored by the AIC of −2885.939 and the size adjusted BIC of −2900.661 (which involves smaller penalties), in combination with the entropy being closer to 1 (entropy = 0.87). All other models were rejected as they did not provide any additional explanatory value for estimating the patterns of change.

**TABLE 2:** Model selection criteria of the Growth Mixture Model (GMM) analysis.

Fit statistics	2 Classes	3 Classes	4 Classes
LogLi (n)	1448.266 (9)	1454.970 (12)	1456.002 (15)
BIC	−2861.323	−**2862.995**	−2853.324
SSABIC	−2889.572	−**2900.661**	−2900.406
Entropy	0.018	**0.869**	0.645
AIC	−2878.531	−**2885.939**	−2882.004
Group size (%) C1	62%	80%	20%
C2	38%	4%	64%
C3		16%	2%
C4			14%
C5			

*LogLi, Log Likelihood; n, number of parameters; BIC, Bayesian Information Criterion; SSABIC, Sample size adjusted Bayesian Information Criterion. Bold values indicate best model fit statistic compared to other classes.*

**FIGURE 4:**
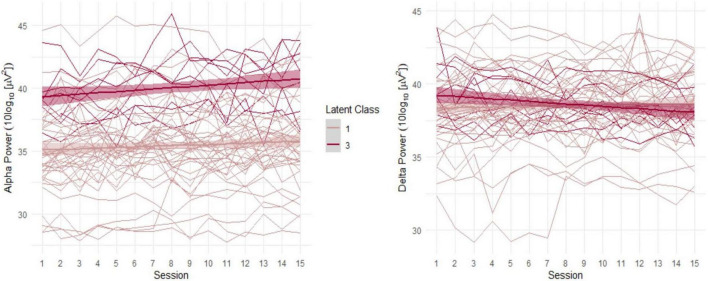
Alpha and delta trajectories in Classes 1 and 3.

As can be seen in Figure 3C, Class 1 (red line) is distinguished by having almost the same level of ADR at the beginning as at the end of the NFB treatment. This class can be considered a *non-responder class*. Class 2 (green line) had a moderate initial ADR with a notable decrease in the slope over time and thus indicates the *Decliner class*. Based on the significant growth factors from the first to the final NFB session that equal an increase in ADR, Class 3 (blue line) will be referred to as the *Responder class* in the following. Further, Class 3 revealed the highest initial ADR. (Although not shown for all classes in Figure 4, there was notable overlap of trajectories, implying that there was considerable fluctuation of individual ADR trajectories within each class.) Classification of individuals based on their most likely class membership resulted in class counts and proportions of 40 (80%) in the non-responder class, two individuals (4%) in the Decliner class, and eight individuals (16%) in the Responder class. The quality of classification can be further indicated by the calculation of posterior probabilities for allocation in a certain class. Individuals of Class 1 had a 94% posterior probability of being correctly classified in the non-responder class, and only 2% posterior probability of being assigned to Class 2, or 4% to Class 3. Similar posterior probabilities were classified for individuals in Class 2 with 90% being in the Decliner class (9% for Class 1 and 0% for Class 3), as well as in Class 3 with 93% being in the Responder class (6% for Class 1 and 0% for Class 2). Even though the 3-class model was favored by the fit indices, unequal class sizes were created. Following statistical justification and interpretability of specifics of class membership, the Decliners, comprising of solely two individuals, were excluded from the remaining analysis, leaving a final sample of 48 individuals that include Responders and non-responders. (We refer the interested reader to Appendix Table 1 for descriptive characteristics of the two individuals of the Decliners class).

**FIGURE 5:**
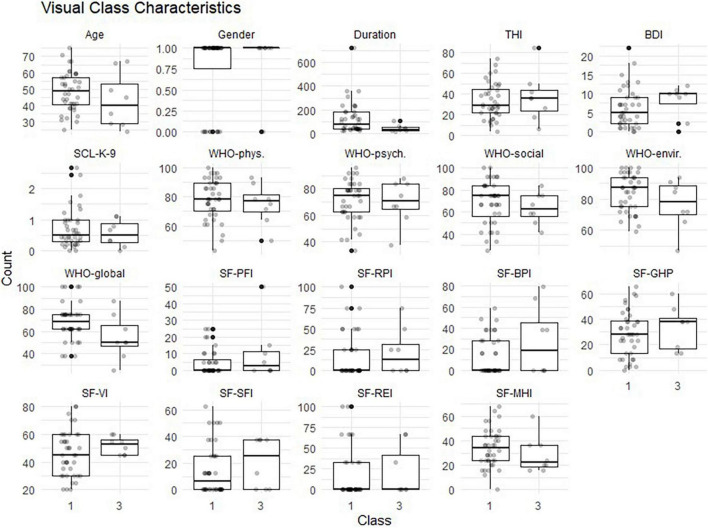
Characteristics of non-responder (class 1) vs. responder (class 3). Sex (male = 1; female = 0); THI, Tinnitus Handicap Inventory; BDI, Beck Depression Inventory; SCL-K-9, Symptom Checklist; WHO, World Health Organization Quality of Life Questionnaire with 4 subscales: phys, physiological, psych, psychological; social, envir; environmental; SF-36, Short Form Health Questionnaire with 8 subscales; PFI, Physical Functioning Index; RPI, Role-Physical Index; BPI, Bodily Pain Index; GHP, General Health Perceptions Index; VI, Vitality Index; SFI, Social Functioning Index; REI, Role-Emotional Index; MHI, Mental Health Index.

In a next step, the two remaining classes, Responder and non-responder, were assessed for indicators of class membership. An overview of the class-specific indicator occurrence is shown in Figure 5. After checking for normal distributions and homogeneous variance, the Firth’s binary logistic regression was performed with class membership (responder vs. non-responder) as dependent variable.

**FIGURE 6:**
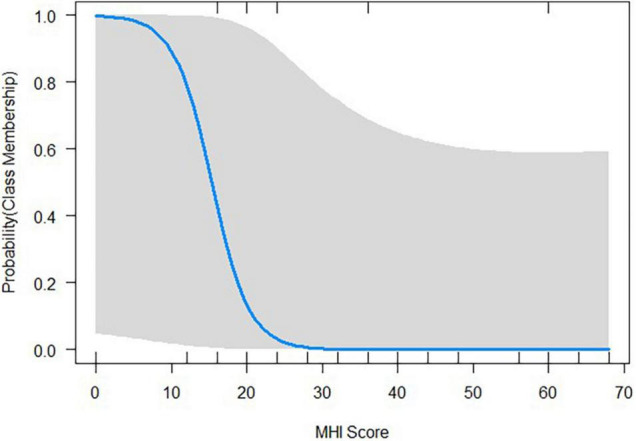
Probability plots illustrating how the class membership probabilities change with the given value of the Mental Health Index (MHI), a subscale of the SF-36 health questionnaire.

Table 3 shows the logistic coefficients for the regression of class membership, with non-responder (dummy coded 0) as the reference class. The Nagelkerke *R*^2^ value was 0.53, and the Hosmer-Lemeshow test value was 0.42. Neither tinnitus distress, depression characteristics, nor general quality of life questionnaire items were significant indicators for class membership. Only the Mental Health index (MHI), a subscale of the SF-36, reached significant negative influence (OR = 0.77, *SE* = 0.12, *p* < 0.05) on class membership. This suggests that each one-unit of increase in the MHI will decrease the log odds of being in the Responder class by 0.266, and the *p*-value indicates that the MHI is significant in determining class membership. We refer the reader to Figure 6 for a graphic representation of the probabilities of group membership. As already mentioned, the significant independent variable is a subscale of the SF-36 health questionnaire. For each subscale, the standard scores were calculated with higher percentage scores indicating either a higher level of functioning or less disability.

**TABLE 3:** Firth’s binary logistic regression with dichotomized dependent variables (0 = non-responder; 1 = responder) of class membership (*n* = 48).

			Class 1: non-responder (*n* = 40)	Class 3: responder (*n* = 8)
			
		Estimate (*SE*)		
	Intercept	Mean	0.91 (0.01)* 0.99 (0.02)*	
	Slope	Mean	0.01 (0.00) 0.01 (0.01)*	
	Variance-covariance	Intercept	0.02	
		Slope	0	
		Intercept-slope	–0.01	
			Regression coefficient (*SE*)	
	Age		Reference class	0.97 (0.04)
	Gender			2.56 (1.29)
	Duration in months			0.98 (0.01)
Tinnitus &	THI			0.99 (0.03)
Depression	BDI			1.15 (0.13)
	SCL			0.24 (1.45)
SF-36	Physical functioning index			1.21 (0.11)
	Role-physical index			0.92 (0.06)
	Bodily pain index			1.01 (0.03)
	General health perceptions index			0.88 (0.08)
	Vitality index			1.24 (0.11)
	Social functioning index			1.11 (0.07)
	Role-emotional index			1.01 (0.03)
	Mental health index			0.77 (0.12)*
WHO-QoL	Physical			1.03 (0.09)
	Psychological			0.99 (0.08)
	Social			0.96 (0.06)
	Environmental			0.92 (0.08)

*THI, Tinnitus Handicap Inventory; BDI, Beck Depression Inventory; SCL, Symptom Checklist; WHO-Qol, World Health Organization Quality of Life Questionnaire; SF-36, Short Form Health Questionnaire; SE, standard error; OR, Odds ratio. 95% Confidence interval in parentheses.*

*Statistically significant at 0.05 level.

Taken together, the results indicate that individuals can be classified into different latent classes based on their 15 weekly EEG recordings taken prior to each NFB training session, and that a 3-class solution provided the best fit among GMM models. Health-related subscale responses on the SF-36 provided the best indicators, whereas the tinnitus distress (THI), depression (BDI), and general quality of life questionnaire (WHO-BREF, SCL-K-9) did not reach significance, providing no additional predictive value for class membership.

## Discussion

According to the definition applied in this report, the only requirement to be classified a Responder is the ability to modify one’s neural oscillations. It should be emphasized here that we are aware of the publication by Gruzelier (2014b), in which it was suggested that a trio of specificities—frequency band, topographical, and outcome specificity—should be fulfilled for a NFB intervention to be labeled successful. While respecting Gruzelier’s consideration to develop a methodological standard in the NFB community, we believed it necessary to highlight the deficits of NFB and therefore to take a step back in the theoretical framework. This is why we chose the statistical tool of growth mixture modeling, which allowed us to quantify the extent of NFB inefficacy in our sample of chronic tinnitus sufferers. In addition, we attempted to predict the probability of being a Responder and whether patterns of change were constrained or dictated by underlying characteristics that had not been previously explored.

By removing subjectivity and making use of all available EEG data, we recovered hidden patients’ trajectories in response to NFB treatment for tinnitus. Based on a representative sample of individuals, we disentangled heterogeneous oscillatory trajectories and identified meaningful subgroups showing similar ADR patterns across 15 weekly sessions. Developing this direction further, we applied a GMM approach that yielded an optimum of three different latent classes, which we named Decliners, non-responders and responders. Decliners exhibited decreases in ADR during the treatment; however, there were only two individuals in this class, which we therefore excluded from further analysis as a precautionary measure. The majority of the participants (80%) were in the non-responder class, defined as those who started and continued the NFB training on the same, unvarying ADR level. Finally, the Responder class comprised eight individuals (16%) who showed the desired increase of the ADR across the NFB training sessions. The findings in our study are generally consistent with previous tinnitus research findings that have shown high variability of treatment response (Kleinjung et al., 2005; Henry et al., 2006; Rossi et al., 2007). Our findings are also in line with NFB research that has indicated large intra-individual differences in EEG patterns (Dohrmann et al., 2007b; Riha et al., 2020) and training trajectories (Riha et al., 2021). Lastly, we found that a certain number of individuals were successful in modifying their EEG activity (Responders), a finding consistent with current literature (Weber et al., 2011; Kouijzer et al., 2013; Enriquez-Geppert et al., 2014; Alkoby et al., 2018).

When looking at the results broken down by latent classes, in Figure 3C, it can be noted that the Responder class (blue line) exhibited the highest initial ADR. However, the existing ADR trajectories are ambiguous as it is unclear whether the ratio change over time is influenced by one or both frequency bands. Separated for alpha and delta power, as shown in Figure 4, it is apparent that the Responder class (Class 3) indeed revealed the desired increase in the alpha-, and decrease in the delta-band. This novel finding caused us to view our past findings from a new perspective, as we had previously only observed change in the alpha-band when including the whole sample in the analysis, without acknowledging the individual trajectories. Moreover, the Responder class’s alpha trajectory started at a significantly higher initial alpha power level compared to the non-responder class (Class 1).

The observed higher initial alpha power in the Responder class follows a concept outlined in the *neural efficiency hypothesis* (Haier et al., 1992; Doppelmayr et al., 2005). According to this hypothesis in the context of NFB, Vernon and colleagues suggest that “if alpha makes completion of a task more efficient by inhibiting non-essential processing, then a greater level of available alpha may enable the individual to inhibit more non-essential activity, which in turn may facilitate performance […]” (Vernon et al., 2009, p. 216). In contrast, low levels of alpha waves reflect a state of excitation (Klimesch et al., 2007). In addition, it has been suggested that alpha enhancement training may lead to higher outgoing connectivity in a neighboring region of the trained area (Hartmann et al., 2014) as it works as a communication vector across cortical areas (Haegens et al., 2015). Expanding on and supporting these lines of thought, our results indicate that individuals with an increased initial alpha power are more likely to be able to actively inhibit irrelevant processes, thus making them more efficient in altering their brain activity during NFB treatment and hence belonging to the Responder class.

The prediction of class membership in the second step, logistic regression analysis, was based on multiple characteristics that represent an approximation to the comprehensive picture of the individual’s general and tinnitus-related quality of life, as well as their health-related wellbeing. The latter construct provided the strongest group of markers, derived from the Short-Form Health Questionnaire (SF-36). Of its eight health-related quality of life dimensions, the MHI represented the strongest predictor. The five item MHI subscale of the SF-36 was developed to measure psychological distress and wellbeing (Ware and Sherbourne, 1992). The subscale’s items relate to anxiety, depression, loss of behavioral or emotional control, and psychological wellbeing. Scoring follows a 0–100 range from low for *feelings of nervousness and depression all of the time* to high for *feeling peaceful, happy, and calm all of the time*. The range of the scale allows for the valid discrimination of psychiatric patients from those with other medical conditions (Berwick et al., 1991). The unique effect of the MHI subscale was very small but may be clinically relevant and is in accordance with the results of other studies indicating the effect of psychological wellbeing and (healthy) mental states on the course and outcome of treatments in various pathologies (Carver et al., 2005; Hasler, 2016; Guidi et al., 2018). It is, however, important to differentiate between the effect on a positive treatment outcome and the ability to learn to self-regulate the brain activity. In this analysis, *poorer* psychological wellbeing was found to predict this ability. The question which then arises is why would poor subjective wellbeing be a prerequisite for the alteration of oscillatory patterns?

Researchers have contrasting views on the influence of psychological factors on the individual ability to modulate EEG patterns. Hammer et al. (2012) have suggested that NFB/BCI performance can only be predicted to a limited extent by psychological parameters. Similar findings were reported by Marxen et al. (2016), who noted that depression has no statistically significant relationship with regulation during fMRI-based NFB training. Given the lack of an association between class membership and depression in our results, we can support these previous findings to some extent. However, taking into consideration the fronto-central position of the electrodes in our study and the coarse spatial resolution of EEG in general, the signal detected cannot assuredly be associated with only the primary auditory cortices; other, non-auditory areas may have contributed as well. The neural correlate for *feelings of nervousness and depression*, as the lower scores of the MHI are defined (Ware and Sherbourne, 1992), can be represented by specific oscillatory patterns in the tinnitus distress network encompassing the ventromedial prefrontal cortex, the parahippocampus, as well as the insula and anterior cingulate cortices (ACC) (Jastreboff, 1990; Lockwood et al., 1998; Mühlau et al., 2006; Moisset and Bouhassira, 2007; Vanneste et al., 2010; De Ridder et al., 2011; Vanneste and De Ridder, 2012). Previous studies have indicated the importance of emotional factors in the experience of tinnitus (Andersson et al., 1999; van der Loo et al., 2011; Joos et al., 2012; Brüggemann et al., 2016; Meyer et al., 2017), and that happiness is associated with temporal parietal regions, while sadness activates limbic and paralimbic structures (Jastreboff, 1990; George et al., 1995). Other scholars found evidence that activities of the paralimbic cortex including the left insula and the rostral and pregenual ACC were of significant predictive value for the change of distress (measured by the THI) in tinnitus retraining therapy (Kim et al., 2016). This was confirmed by resting-state EEG data indicating that the level of distress is further correlated with alpha oscillation over these areas (Vanneste et al., 2010). A recent report supports the notion that if the oscillatory activity of the ACCs is insufficient prior to the initial fitting and wearing of hearing aids in the treatment for tinnitus, the phantom perception cannot be improved by the devices (Han et al., 2020). These latter findings accord with our results and guide the attention back to the described top-down inhibiting processes of alpha oscillations. As previously mentioned, contemporary research on NFB has indicated that higher resting-state alpha is associated with increased probability of learning to modify the targeted brain waves during treatment (Klimesch et al., 2007; Gruzelier, 2014a; Wan et al., 2014).

Furthermore, the insula and the ACC are key regions of the salience network (SN) which mediates filtering and detecting salient stimuli (Seeley et al., 2007; Menon, 2015). Simply put, the SN first filters the constant stream of incoming stimuli according to their perceptional features (Peters et al., 2005). As Menon (2015) states, stimuli are more likely to be perceived as salient if they “include deviants embedded in a constant stream, surprising stimuli, and stimuli that are pleasurable and rewarding, self-relevant, or emotionally engaging” (p. 597). Once a salient stimuli is detected, the network’s robust connections recruit other brain networks and facilitate access to attention and working-memory resources (Sridharan et al., 2008). A shift of attention from external to internal processes is suggested, resulting in the representation of a subjective and conscious state, as well as the emotional value of the external stimuli (Seeley et al., 2007; Goulden et al., 2014). Thus, the SN is further associated with internally oriented mental processes and interoceptive awareness, which is associated with autonomic processes such as heartbeat, skin conductance, and respiration. In the context of tinnitus, it has been suggested that a persistent state of awareness may lead to the misattribution of salience to a stimulus, and that this could explain the genesis and maintenance of a conscious auditory percept to a non-existent sound (Rinne et al., 2009; Sadaghiani et al., 2009; De Ridder et al., 2014). Driven by the persistent awareness, the SN seems to act as a multisensory integration site of different tinnitus aspects and attributes, thus making it a core modulator of tinnitus-related distress and subjective wellbeing (van der Loo et al., 2011; De Ridder et al., 2014; Shi et al., 2018).

While our interpretation of results builds on the approach presented in this report, the neural component of conscious, health-related wellbeing and its oscillatory activity or fluctuations could have a number of other potential causes. The challenge in interpreting these effects lies in determining whether they are associated with the generation and chronification of the tinnitus percept, or whether they are associated with tinnitus-related reactions and/or compensations on the individual level. Disentangling wellbeing into its constituent parts and considering our data, we cannot clearly differentiate between tinnitus-related and health-related wellbeing, nor can we identify which of these potential mechanisms might be most relevant. Since the dynamics of neural oscillations reflect perceptual, sensory, cognitive and emotional events, the precise details of these mechanisms warrant further attention. However, our results supported the general assertion that mental wellbeing—as derived from the items of the MHI in this analysis—is decisive for the course and outcome of an NFB treatment. Indeed, the effects were determined at both ends of the defined continuum, at levels of both low and high wellbeing.

### Limitations

The inefficacy problem, as one ambiguity concerning NFB, has been the focus of this report. However, other pertinent points in this treatment approach remain to be considered. The most important points are first, that the underlying mechanisms of NFB are not entirely understood and the discussion of its effects is ongoing (Fovet et al., 2017; Schabus, 2017; Thibault et al., 2017; Witte et al., 2018). Second, the demands of temporal expenditure for both participants and clinicians in NFB mean that more distinct and clinically applicable predictors for the ability to learn the regulation of brain activity are urgently needed. The ultimate point we mention here refers to clinical study protocols; for example, duration and frequency of training, feedback modality, and the lack of a blinded control or placebo group (Vernon et al., 2009; Cortese et al., 2016; Omejc et al., 2019; Jensen et al., 2020). Detailed information on all aspects of the discussion orbiting NFB are unfortunately beyond the scope of this report and we refer to existing publications (Gruzelier, 2014b; Rogala et al., 2016; Hampson et al., 2019). It would be certainly not correct to view the limiting factors exclusively from the aspect of NFB, but rather it is necessary to raise awareness about the inferences in this report. Our results are restricted to oscillatory patterns prior to several NFB training sessions and are sensitive to and dependant on the variation of the sample. Additionally, the number of individuals in our sample who underwent a longitudinal clinical NFB trial would be considered moderate yet, for analysis in the GMM framework, it is in the lower ranges. Hence, the observations and inferences presented here can only be treated as qualitative on incidental results. Access to data collected on a larger, more diverse group would give better estimates of this potential dependence. Additionally, we must ask future researchers to consider and include intervention-specific outcomes (Hall et al., 2019), such as hearing thresholds, openness to technical novelties, measures from MRI examinations, and other clinically applicable measures as possible predictors for failure to control in their analysis.

## Conclusion

Our findings support the idea that the treatment of tinnitus with NFB is a promising technique. However, individuals displayed heterogeneous trajectories during the training while low levels of health-related wellbeing seemed to be a prerequisite for the ability to modify the brain activity in the desired direction. In addition, our efforts to identify individual trajectories and thus bring clarity to the existing literature through the application of GMM would not have been possible if we had treated the individuals in our study of NFB treatment for tinnitus as a single group and used mean level data as adopted in previous studies. Our data-driven approach in this report presents a step toward enabling the translation of scientific findings into suitability for everyday medical practice, bettering the definition of tinnitus “subtypes” in heterogeneous treatment responses, and hence supporting precision medicine. To help achieve the vision of NFB becoming part of precision medicine, both the technology and the general understanding of tinnitus-specific brain activity require continued research, with special consideration being given to health-related wellbeing.

## Data Availability Statement

The data is not yet openly available due to ongoing analysis. Thus, we regret that the data is not publicly accessible at this time. Requests to access these datasets should be directed to the corresponding author.

## Ethics Statement

The studies involving human participants were reviewed and approved by the Kantonale Ethikkommission Project KEK-ZH-Nr. 2014-0594. The patients/participants provided their written informed consent to participate in this study.

## Author Contributions

DG carried out the neurofeedback training and the data acquisition. CR designed the model and the computational framework, analyzed the data, and wrote the manuscript. MM provided comments on the final draft. TK helped in supervise the project. All authors contributed to the article and approved the submitted version.

## Author Disclaimer

The views expressed in this article are those of the authors and not necessarily those of the funders.

## Conflict of Interest

The authors declare that the research was conducted in the absence of any commercial or financial relationships that could be construed as a potential conflict of interest.

## Publisher’s Note

All claims expressed in this article are solely those of the authors and do not necessarily represent those of their affiliated organizations, or those of the publisher, the editors and the reviewers. Any product that may be evaluated in this article, or claim that may be made by its manufacturer, is not guaranteed or endorsed by the publisher.

## Footnotes

1
http://www.tinnet.tinnitusresearch.net/

## Appendix

**TABLE A1:** Demographic, health, and tinnitus characteristics of the two individuals of the decliner class.

		Decliner #1	Decliner #2
	Age	36	37
	Gender	Male	Male
	Tinnitus duration (months)	36	8
Tinnitus and	THI	18	32
depression	BDI	3	3
	SCL	0.55	0.33
SF-36	Physical functioning index	5	0
	Role-physical index	0	50
	Bodily pain index	16	45
	General health perceptions index	23	18
	Vitality index	35	40
	Social functioning index	12.50	0
	Role-emotional index	0	0
	Mental health index	12	8
WHO-QoL	Physical	85.71	71.43
	Psychological	79.17	79.17
	Social	66.67	100
	Environmental	90.63	75

*THI, Tinnitus Handicap Inventory; BDI, Beck Depression Inventory; SCL, Symptom Checklist; WHO-Qol, World Health Organization Quality of Life Questionnaire; SF-36, Short Form Health Questionnaire.*
